# EGFR-TKIs治疗非小细胞肺癌EGFR罕见突变的研究进展

**DOI:** 10.3779/j.issn.1009-3419.2019.09.07

**Published:** 2019-09-20

**Authors:** 文兴 杜, 杨 沃, 通 卢, 元勇 王, 文捷 矫

**Affiliations:** 266071 青岛，青岛大学附属医院胸外科 Department of Thoracic Surgery, the Affiliated Hospital of Qingdao University, Qingdao 266071, China

**Keywords:** 表皮生长因子受体, 罕见突变, 酪氨酸激酶抑制剂, 肺肿瘤, Epidermal Growth Factor Receptor, Uncommon Mutation, Tyrosine Kinase Inhibitor, Lung neoplasms

## Abstract

肺癌是目前最常见的癌症，也是导致癌症死亡的首要原因。非小细胞肺癌（non-small cell lung cancer, NSCLC）占85%以上，且高达50%的亚洲NSCLC患者携带表皮生长因子受体（epidermal growth factor receptor, *EGFR*）基因突变。研究证明，伴有*EGFR*突变的NSCLC患者接受表皮生长因子受体-酪氨酸激酶抑制剂（epidermal growth factor receptor-tyrosine kinase inhibitors, EGFR-TKIs）治疗能获得更好的生存结果。然而，因为*EGFR*罕见突变相对治疗效果较差，会对研究结果带来负面影响，所以大部分研究EGFR-TKIs疗效的临床试验都不包含罕见突变患者，另外*EGFR*罕见突变本身就少见，就导致临床试验中这部分患者数量较少。由于*EGFR*罕见突变样本量少且具有高度异质性，EGFR-TKIs对*EGFR*罕见突变患者的疗效仍然不清楚。本文就*EGFR*罕见突变与EGFR-TKIs的疗效关系进行综述，为携带*EGFR*罕见突变的NSCLC患者合理选择治疗方式提供指导和建议。

## Abstract (en)

Lung cancer is the most common cancer and the leading cause of cancer death. Non-small cell lung cancer (NSCLC) represents over 85% of all lung cancers, and up to 50% of Asian NSCLC patients harboring epidermal growth factor receptor (*EGFR*) gene mutations. A number of studies have consistently demonstrated that uncommon *EGFR*-mutated NSCLC patients treated with EGFR-tyrosine kinase inhibitors (EGFR-TKIs) can achieve better survival outcomes. However, because uncommon *EGFR* mutations are generally associated with reduced sensitivity to EGFR-TKIs, which will bring a negative impact on the result of the study, the majority of clinical trials investigating the efficacy of EGFR-TKIs have included only patients with common *EGFR* mutations. In addition, uncommon *EGFR* mutations are rare in themselves, leading to the small number of such patients enrolled in these trials. Due to the small number and highly heterogeneous sensitivity of uncommon *EGFR* mutations, the efficacy of EGFR-TKIs in patients harboring uncommon *EGFR* mutations remains elusive. This article reviews the efficacy of EGFR-TKIs in patients with uncommon *EGFR* mutations, and give some reasonable advice about the selection of treatments for patients with NSCLC who harbor uncommon *EGFR* mutations.

## 1 前言

肺癌是目前最常见的癌症，也是导致癌症死亡的首要原因，其中非小细胞肺癌（non-small cell lung cancer, NSCLC）占85%以上^[1, 2]^。随着靶向癌基因的发现和对NSCLC进展分子驱动因素的研究不断发展，NSCLC患者的生存结果不断改善。2004年首次发现NSCLC患者细胞内发生表皮生长因子受体（epidermal growth factor receptor, *EGFR*）基因突变^[3]^，大约10%-15%高加索患者和高达50%的亚洲NSCLC患者携带*EGFR*突变^[4-6]^，其中女性多发，多数患者不吸烟或轻度吸烟，组织病理学以肺腺癌为主^[7]^。我国最新的指南推荐EGFR-酪氨酸激酶抑制剂（EGFR-tyrosine kinase inhibitors, EGFR-TKIs）作为*EGFR*突变晚期NSCLC患者的一线标准治疗。

多项临床研究^[8-14]^明确证实，相比于传统化疗，伴有*EGFR*突变的NSCLC患者对EGFR-TKIs单药治疗更敏感，EGFR-TKIs治疗的总有效率（overall response rate, ORR）大于70%，中位无进展生存期（progression-free survival, PFS）长达9.6个月-18.9个月，中位总生存期（overall survival, OS）达21.6个月-34.1个月。然而，因为*EGFR*罕见突变相对治疗效果较差，会对研究结果带来负面影响，所以大部分研究EGFR-TKIs疗效的临床试验都不包含罕见突变患者^[15, 16]^，另外*EGFR*罕见突变本身就少见，就导致临床试验中这部分患者数量较少。由于*EGFR*罕见突变患者样本量少且具有高度异质性，EGFR-TKIs对*EGFR*罕见突变患者的疗效至今仍然不清楚。随着基因检测技术的快速发展，*EGFR*罕见突变的检出率会不断增加，更清楚地了解这部分患者对各种TKIs治疗的敏感性有着重要意义。本文就*EGFR*罕见突变类型与EGFR-TKIs的疗效关系进行综述，为携带*EGFR*罕见突变的晚期NSCLC患者合理选择治疗方式提供指导和建议。

## 2 EGFR的生物学特征和*EGFR*基因突变

表皮生长因子受体（EGFR/her1/erbB1）属于酪氨酸激酶受体家族，也被称作HER家族或erbB家族，这个家族其他成员包括HER-2（Neu, ErbB2）、HER-3（ErbB3）和HER-4（ErbB4）。EGFR是一种跨膜受体，结构上分为胞外区（细胞外配体结合区）、跨膜区、胞内区（细胞内酪氨酸激酶区）三部分。当EGFR胞外区与相应配体结合后，细胞内的酪氨酸激酶区被激活，导致自身磷酸化，从而为多种下游分子提供停泊位点，启动下游信号转导通路^[17]^。EGFR-TKIs就是一类作用于细胞内酪氨酸激酶区的小分子药物，通过阻断EGFR信号通路，从而抑制NSCLC的生长、转移和侵袭^[18]^。

*EGFR*基因位于第七号染色体短臂（7p12-14）上，长约118 kb，由28个外显子组成^[17]^。编码EGFR激酶域的基因位于18号-24号外显子，而NSCLC患者*EGFR*突变主要发生在18号-21号外显子，当编码EGFR激酶域的基因发生突变时会导致受体激酶活性增加，所以*EGFR*基因突变常被称为*EGFR*激活突变^[19, 20]^。其中19号外显子缺失突变和21号外显子L858R点突变，这两种突变类型约占所有*EGFR*突变的90%，被称为*EGFR*常见突变或*EGFR*经典突变，且这两种突变都会导致酪氨酸激酶域的激活（与对EGFR-TKIs的敏感性相关），所以这两种突变又常被称为*EGFR*敏感突变^[21]^。而其他*EGFR*突变类型因突变率低，一般统称为*EGFR*罕见突变或*EGFR*不常见突变^[22]^，包括G719突变、E709X突变、19号外显子插入突变、20号外显子插入突变、T790M点突变、S768I点突变、L861Q点突变等。

## 3 18号外显子突变

18号外显子突变约占所有*EGFR*突变的3%-4%，包括点突变和缺失/插入突变，其中点突变包括G719X及其变体（G719A/G719S/G719C等）和E709X，缺失突变包括delE790_T710insD等。

G719X点突变是18外显子突变最常见的突变类型，约占*EGFR*罕见突变的20%^[23]^。Kobayashi等^[24]^的研究提出，与第一代TKIs相比，G719X突变对第二代TKIs（阿法替尼或来那替尼）的敏感性更强。大量临床研究结果支持这一观点（表 1）^[10, 22, 23, 25-29]^，第一代TKIs治疗G719X突变的NSCLC患者的反应较好（ORR：14%-53.3%，中位PFS：5.98个月-11.6个月，中位OS：16.4个月-25.2个月），但相比于经典突变敏感性还是略低; 而第二代TKIs治疗G719X突变的NSCLC患者的总有效率为75%-77.8%，中位无进展生存期为12.1个月-13.8个月，中位总生存期为26.9个月，敏感性与经典突变相似，可见G719X突变对第二代TKIs的敏感性要更高。目前尚缺乏G719X突变对第三代TKIs敏感性的临床研究。所以，推荐携带G719X突变的晚期NSCLC患者首选第二代EGFR-TKIs作为一线治疗。

**1:** 携带EGFR18号外显子基因突变的患者对EGFR-TKIs的疗效 Activity of EGFR-TKIs in patients harboring exon 18 mutation

Study	Mutation (s) included	N	EGFR-TKIs used	ORR	mPFS (mo)	mOS (mo)	References
Wu JY, 2011	Single and complex G719X mutations	15	G, E	53.3%	8.1	16.4	[28]
NEJ-002, 2014	Single G719X mutations	7	G	14%	NR	NR	[10]
Chiu CH, 2015	Single G719X mutations(78) G719X + L861Q(9) G719X + S768I(10)	97	G, E	36.8% 88.9% 50.0%	6.3 NR NR	NR NR NR	[27]
Xu J, 2016	Single G719X	14	G, E, I	42.9%	5.98	19.81	[26]
Zhang Y, 2017	Single G719X(14), complex G719X mutations(8)	22	G, E, I	22.7%	7.6	NR	[25]
Tu HY, 2017	Single G719X(12), complex G719X mutations(4)	16	G, E	50.0%	11.6	25.2	[23]
Sequist LV, 2010	Complex G719X mutations	4	N	75%	12.1	NR	[29]
Yang JC, 2015	Single G719X(8), complex G719X mutations(10)	18	A	77.8%	13.8	26.9	[22]
Wu JY, 2016	Complex E709X mutations	20	G, E	50%	6.2	29.3	[30]
Wu JY, 2016	delE790_T710insD	5	G, E	0%	2.3	NR	[30]
G: Gefitinib; E: Erlotinib; A: Afatinib; I: Icotinib; N: Neratinib; NR: not reported; ORR: overall response rate; mPFS: median progression-free survival; mOS: median overall survival; mo: months.							

E709X点突变占所有*EGFR*突变的比例小于0.5%^[23]^。由于检测技术的限制，E709X突变的发生率偏低，又因其多与其他突变联合出现，所以有关E709X突变与TKIs疗效关系的系统研究较少。Wu等^[30]^的研究证明（表 1），E709X突变对第一代TKIs敏感，但敏感性比*EGFR*经典突变略低（ORR: 50.0% *vs*. 74.1%）。虽然没有大样本临床试验证明E709X与第二代TKIs疗效的关系，但Heigener等^[31]^的研究发现了一个有趣的现象，经一线治疗进展后的10例G719X突变患者，再用阿法替尼治疗失败时间为2.6个月，而携带E709X突变的4例患者治疗失败时间达12.2个月，这个结果提示E709X突变可能对第二代TKIs阿法替尼更敏感。现在的问题是E709X突变患者接受不同EGFR-TKIs治疗获益是否相同，则需要大样本量的临床试验来进一步验证。

在所有18号外显子缺失突变中，delE790_T710insD突变是最为常见的一种类型，约占总*EGFR*突变的0.16%^[28]^。目前暂时缺乏delE790_T710insD突变与第一代TKIs疗效关系的系统研究，Kobayashi等^[32]^的临床前研究则认为该突变是对EGFR-TKIs最不敏感的18号外显子突变。后来Wu等^[30]^报道的5例delE790_T710insD突变患者对第一代TKIs治疗的反应均较差（表 1）。但最近1例个案报道^[33]^表明该突变患者接受阿法替尼治疗两个月后，影像学显示病灶明显缩小，这提示我们delE790_T710insD突变患者可能对第二代TKIs敏感。其他更少见的18号外显子基因突变对EGFR-TKIs有反应的包括：V689M、S720P/F、P699S、N700D、E709Q、G721A、V740A、L718P等; 对EGFR-TKIs耐药的包括：E711K、G721D、G729R、I744M、K708M、L692P、L703F、L703P等; 敏感性不确定的有：I715S、L718P、L688P、P694X、G724S等^[34]^。

## 4 19号外显子突变

19号外显子突变包括一种经典突变即缺失突变（约占所有*EGFR*突变的50%）^[35]^，其他则是罕见突变包括不常见缺失突变、插入突变和点突变等。

19号外显子缺失突变主要是指保守模体LREA（残基747-750）缺失（属于经典突变类型），其中最常见的一种类型是delE746-A750（66.1%），其次是delL747-P753insS（56.8%）; 但仍存在几种其他不常见缺失突变类型，不同缺失突变类型对EGFR-TKIs的敏感性并不相同^[36, 37]^。Kuei-Pin等^[36]^的研究提出，在不同氨基酸位置缺失的NSCLC患者中，EGFR-TKI治疗的反应可能不同，19号外显子非LRE缺失的患者对EGFR-TKIs的反应比LRE缺失的患者更差。这一研究提示，并不是所有19外显子缺失突变都对EGFR-TKIs有很好的敏感性，随着测序技术的进步，针对19号外显子非LRE缺失的患者应如何用药，将会成为下一步研究的一个重要方面。

19号外显子插入突变即使在罕见突变中也较少见，约占所有*EGFR*突变的0.2%。最早有病例报道^[38]^表示1例伴19外显子插入突变NSCLC患者对第一代TKIs吉非替尼敏感，无进展生存期为10.4个月，总生存期为29.5个月。之后He等^[39]^的体外试验表明，携带19外显子插入突变的Ba/F3细胞对第一、二代TKIs敏感，但比经典突变的敏感性低。且Lin等^[40]^的临床试验也证明了这一观点（表 2），3例19号外显子插入突变患者对TKIs治疗具有敏感性，结果为1例部分缓解，1例病情稳定，1例病情进展。结合公开数据分析得出，19外显子插入突变患者接受EGFR-TKI治疗的敏感性低于经典突变患者（ORR: 56%，中位PFS: 10.4个月）。由于该突变发生率低，19号外显子插入突变与TKIs疗效的关系仍需要更多大样本量的系统研究证实。

**2:** 携带EGFR 19号外显子基因突变的患者对EGFR-TKIs的疗效 Activity of EGFR-TKIs in patients harboring exon 19 mutation

Study	Mutation (s) included	N	EGFR-TKIs used	ORR	mPFS (mo)	mOS (mo)	References
Uruga H, 2010	exon 19 insertion	1	G	NR	10.4	29.5	[38]
Lin YT, 2017	exon 19 insertion	18	G, E, A	56%	10.4	NR	[40]
Lin YT, 2017	exon 19 insertion	6	G, E, A	NR	NR	24	[40]

其他19号外显子罕见突变类型报道较少，它们对不同EGFR-TKIs的敏感性也各不相同，对TKIs有反应的包括：P733L/S、N756D、E758G、N756D、E758G等; 对TKIs耐药的包括：D761Y/N、E746V、L747S/P、R748W、V742A等; 敏感性不确定的有：G735S、P733L、S752Y、T751I、V738F、V742A等^[34]^。

## 5 20号外显子突变

20号外显子突变包括插入突变，T790M点突变和S768I点突变等。20号外显子插入突变是*EGFR*罕见突变中最常见的一种突变类型，约占*EGFR*罕见突变的30%，占所有*EGFR*突变的4.8%-12%，已检测出64种不同的突变亚型，其中最常见的一种是D770_N771>ASVDN（21%）^[23, 41, 42]^。突变插入的位置和大小变化很大，从3 bp-12 bp不等，这可能是该突变患者对EGFR-TKIs的敏感性高度异质的原因^[37, 41, 43]^。既往研究^[22, 23, 41]^表明（表 3），当接受第一、二代EGFR-TKIs治疗时，携带20号外显子插入突变患者的总有效率为0%-11%，中位无进展生存期为2个月-3个月，总生存期与野生型EGFR患者相似。可见携带20外显子插入突变的患者对第一、二代TKIs药物反应较差，可将化疗作为该突变患者的一线治疗方案。而最近Lee等^[44]^的临床前研究表明，第三代EGFR-TKI奥西替尼对包括H773insH突变体在内的20号外显子插入突变细胞和野生型EGFR细胞均有较强的杀伤作用。随后Fang等^[42]^的临床研究首次证实了奥西替尼在20号外显子插入突变的晚期NSCLC患者中具有良好的抗肿瘤活性（中位PFS：6.2个月）。由此可知，20号外显子插入突变患者接受第三代TKIs奥西替尼治疗受益可能会更好，但仍需要更多临床试验验证。近来有体外研究^[45]^用阿法替尼联合西妥昔单抗治疗20外显子插入突变，结果表明，对于EGFR A767_V769dupASV和EGFR Y764_V765insHH，西妥昔单抗或阿法替尼单独治疗对肿瘤的形成无明显抑制作用; 然而，阿法替尼联合西妥昔单抗治疗对肿瘤生长有显著抑制作用，且无明显的体质量减轻和皮疹。这提示我们当TKIs治疗反应欠佳时，可以考虑联合其他药物共同使用，或许会有更好的疗效。

**3:** 携带EGFR 20号外显子基因突变的患者对EGFR-TKIs的疗效 Activity of EGFR-TKIs in patients harboring exon 20 mutation

Study	Mutation(s) included	N	EGFR-TKIs used	ORR	mPFS (mo)	mOS (mo)	References
Tu HY, 2017	exon 20 insertion	12	G, E	0%	3	12.5	[23]
Riess JW, 2018	exon 20 insertion	5	NS	0%	3.5	NR	[41]
Yang JC, 2015	exon 20 insertion	23	A	8.7%	2.7	9.2	[22]
Fang W, 2019	exon 20 insertion	6	O	66.7%	6.2	NR	[42]
Xu JL, 2016	Del-19 or L858R + T790 M mutations	9	G, E, I	22.2%	1.94	16.89	[26]
Zhang B, 2018	T790M/20insertion+others	12	NS	8.3%	1.4	NR	[47]
Yang JC, 2015	Single de-novo T790M(3), Complex de-novo T790M mutations(11)	14	A	14.3%	2.9	14.9	[22]
Mok TS, 2017	T790M	279	O	71%	10.1	NR	[49]
Wu JY, 2011	S768I complex mutations	4	G, E	75%	NR	NR	[28]
Peng L, 2014	S768I + L858R	1	G	0%	6.0	6.5	[51]
Chiu CH, 2015	Single S768I; S768I + G719X	7 10	G, E	33.3% 50.0%	NR	NR	[27]
Levantakos K, 2016	Single S768I(1); complex S768I mutations (3)	4	E	25%	NR(3-20)	NR(5-51)	[50]
Zhang Y, 2017	Single S768I(4), complex S768I mutations(7)	11	G, E, I	27.3%	8.0	NR	[25]
Yang JC, 2015	Single S768I(1); complex S768I mutations(7)	8	A	100%	14.7	NR	[22]
Passaro A, 2019	S768I complex mutations	16	G, E, A	56.2%	NR	NR	[6]
O: osimertinib; NS: not specified.							

原发性T790M突变发生率约占所有EGFR的3%^[46]^，该突变对第一、二代EGFR-TKIs的敏感性偏低。有研究证明（表 3）^[22, 26, 47]^，携带T790M突变的患者接受第一、二代EGFR-TKIs治疗后中位无进展生存期为1.4个月-2.9个月，中位总生存期为14.9个月-16.89个月; 即使与敏感突变共存，复合T790M突变对第一、二代TKIs的敏感也不高。T790M突变还被认为是第一、二代EGFR-TKIs获得性耐药机制中最常见的突变类型，对TKIs获得性耐药的患者中约50%存在继发性T790M突变^[48]^。但随着第三代TKIs的出现，继发性T790M突变患者有了更好的生存结果，Mok等^[49]^的Ⅲ期研究比较了奥西替尼和传统化疗对T790M耐药突变NSCLC患者的疗效，研究结果显示，奥西替尼组明显优于传统化疗组（ORR：71% *vs* 31%，中位PFS：10.1个月*vs* 4.4个月），且对中枢神经系统转移患者的疗效更好。虽然已证实奥西替尼对T790M突变具有很好的疗效，但其治疗一段时间后会再次出现耐药的情况，而对于奥西替尼发生耐药的后续治疗仍有待进一步的研究。

S768I点突变可以单独发生，但常与其他*EGFR*敏感突变共同出现，约占所有*EGFR*突变的2%-3%。Leventakos等^[50]^的研究证实S768I突变对第一代EGFR-TKIs敏感，但敏感性差异很大（PFS：3个月-20个月，OS：5个月-51个月），且多项临床研究证明（表 3）^[6, 22, 25, 27, 28, 51]^，S768I点突变对第一代TKIs的敏感性略低于经典突变（ORR：50%，中位PFS：6个月-8个月)。Banno等^[52]^的体外研究指出，相比于第一、三代TKIs，S768I突变对第二代TKIs阿法替尼有更高的敏感性，临床研究结果也支持这一观点（表 3）^[22]^，S768I突变患者接受阿法替尼治疗的总有效率为100%，中位无进展生存期为14.7个月，远比第一代TKIs的敏感性好。因此，S768I突变患者选择第二代TKIs治疗获益会更大，但也应注意个体的敏感性差异。

20号外显子其他罕见突变对TKIs敏感的突变有：V765A、T783A、V774A、S784P、R776C、R776H、V765M、G779C、G779F、G779S、T783A、T783I、L798F、L798H、K806E、Q812R、L814P等; 不敏感的突变有：D770_N771 ins NPG/D770_N771insNPG、N771GY delN771insGY、N771dupN A767_V769dupASV、H773_V774insH，、770_771ins VDSVDNP等; 突变敏感性不确定的有：G810S、L792P、T783A等^[34]^。

## 6 21号外显子突变

21号外显子突变除包括一种经典突变即L858R点突变（约占所有*EGFR*突变的40%）之外^[35]^，还包括一部分对TKIs反应不同的点突变。Leduc等^[53]^的研究表明，这些点突变多数对EGFR-TKIs的敏感性要比L858R点突变低（中位PFS：4.5个月*vs* 10.4个月，*P*=0.003;OS：12.2个月*vs* 16.9个月，*P*=0.04）。21号外显子突变除L858R点突变以外最常见的就是L861Q点突变，约占所有*EGFR*突变的1%^[54]^。多项研究^[10, 26, 27, 55-57]^表明（表 4），L861Q突变对第一代EGFR-TKIs敏感（ORR：33.3%-46.7%，PFS：5.16个月-8.90个月，OS：14.49个月-21.98个月），但敏感性不如经典突变，而且复合L861Q突变对一代TKIs的敏感性比L861Q单突变要好得多（ORR: 88.9% *vs* 39.6%）。Yang等^[22]^的分析表明L861Q突变患者接受阿法替尼治疗的总有效率为56.3%、无进展生存期为8.2个月、总生存期为17.1个月。可见第二代TKIs治疗L861Q突变患者的疗效比第一代TKIs更好，这与18外显子G719X突变，20外显子S861I突变具有相似性。携带L861Q突变的患者对第三代TKIs的敏感性至今没有系统临床研究，但Masuzawa等^[58]^的体外试验发现，复合T790M的L861Q突变对第三代EGFR-TKIs的敏感性要比复合T790M的经典突变高10倍-100倍，这提示我们第三代TKIs对伴有L861Q突变患者可能有不错的疗效，期待进一步的临床试验验证。目前还是认为L861Q突变患者选择二代TKIs阿法替尼作为首选治疗获益更大。

**4:** 携带EGFR 21号外显子基因突变的患者对EGFR-TKIs的疗效 Activity of EGFR-TKIs in patients harboring exon 21 mutation

Study	Mutation (s) included	N	EGFR-TKIs Used	ORR	mPFS (mo)	mOS (mo)	References
Leduc C, 2017	L861Q/other uncommon mutation	27	NS	NR	4.5	12.2	[53]
Keam B, 2014	Single L861Q (3), complex L861Q mutations (1)	4	G, E	50%	NR(0.8-7.9)	NR (0.9-26.2)	[55]
NEJ-002, 2014	Single L861Q	3	G	33%	NR	NR	[10]
Chiu CH, 2015	Single L861Q Complex L861Q	57 9	G, E	39.6% 88.9%	8.1 NR	NR NR	[27]
Xu J, 2016	Single L861Q	15	G, E, I	46.7%	8.9	21.98	[26]
Klughammer B, 2016	Single L861Q	3	E	33.3%	NR	NR	[56]
Pilotto S, 2018	Single L861Q	5	G, E	40%	5.16	14.49	[57]
Yang JC, 2015	Single L861Q (12), complex L861Q mutations(4)	16	A	56.3%	8.2	17.1	[22]

其他21外显子罕见突变发生率低，它们对EGFR-TKIs有反应的有：A871V/G、E868G、L836C、L838P、L839T、L861Q/R、L863D等; 耐药的有：G857E、V851I、A859T、G863S、G874S、K860E、L862Y、N826Y、N826S等; 敏感性不确定的有：E866K、H835L、H870R、I853T、T847I、V851X等^[34]^。

## 7 复合突变

复合突变是指在NSCLC患者肿瘤细胞中同时检测到两种或两种以上不同类型的*EGFR*突变，约占所有*EGFR*突变的2.75%-14%^[23, 32, 51]^。*EGFR*复合突变大致可分为三类：双经典突变、经典突变与罕见突变共存，以及不同罕见突变共存。Xu等^[26]^的研究表示，与伴经典突变的T790M复合突变相比，经典突变与其他罕见突变共存的复合突变对TKIs的敏感性更好（ORR: 55.6% *vs* 22.2%)，且双经典突变对TKIs的敏感性是所有复合突变中最好的（ORR: 71.4%）。并且有研究证实^[59, 60]^，接受EGFR-TKIs治疗后，经典突变和罕见突变共存的复合突变比仅含罕见突变的复合突变的预后更好。所以，总体上来说，三类复合突变对EGFR-TKIs的敏感性由强到弱依次是：双经典突变，经典突变伴罕见突变的复合突变，仅含罕见突变的复合突变。且经典突变伴耐药罕见突变的复合突变比经典突变伴敏感罕见突变的复合突变的反应差，这提示*EGFR*复合突变对TKIs的疗效可能受到各伴随突变对TKIs敏感性的影响，例如，携带T790M耐药突变的患者预后普遍比较差。然而，复合20外显子插入突变并没有表现出这种规律，过去常认为20号外显子插入突变是一种耐药突变^[37]^，最近有一项大型回顾性研究^[6]^却给出了不同的答案，复合20外显子插入突变与其他复合突变具有相似的无进展生存期和总生存期。原因可能是由于20外显子插入突变具有高度异质性，不同的突变亚型对TKIs的反应不同，这就需要更多研究来进一步验证。因为有关罕见突变的数据多来源于小样本回顾性研究，所以研究结果受到个体性差异的影响较大，仍有待大样本量、前瞻性的临床研究证明上述观点的准确性。

## 8 其他治疗

当*EGFR*罕见突变患者对EGFR-TKIs治疗不敏感时，及时改变治疗策略对这部分患者具有重要意义。现有的其他治疗方法主要包括化疗、免疫治疗和抗血管生成治疗等。Watanabe等^[10]^对NEJ002试验的分析表明，*EGFR*罕见突变组和常见突变组接受化疗后的生存结果相似（中位PFS：5.9个月*vs* 5.4个月，*P*=0.847;中位OS：22.8个月*vs* 28个月，*P*=0.358），对TKIs耐药的罕见突变患者来说，推荐化疗作为其首选的治疗方案。最近一项Ⅲ期临床试验（NEJ009）结果证明^[61]^，在*EGFR*常见突变患者中，EGFR-TKIs联合化疗组的总生存期比TKIs单药治疗组要好得多，联合治疗组的中位总生存期竟长达52.2个月。虽然该试验只包括常见突变患者，但总结之前的研究可知，部分罕见突变患者（如G719X突变和L861Q突变等）对化疗和TKIs单药治疗的疗效均与经典突变类似或者是略差，提示我们*EGFR*罕见突变患者接受TKIs加化疗的联合治疗也可能获得令人惊喜的结果，期待未来进一步的临床研究能够验证这一观点。

目前，暂时缺乏有关*EGFR*罕见突变与免疫抑制剂疗效关系的临床研究数据。但有越来越多的证据表明，与EGFR野生型NSCLC患者相比，*EGFR*突变患者对以程序性死亡受体-1（programmed death-1, PD-1）及其程序性死亡配体-1（programmed death ligand-1, PD-L1）为靶点的免疫抑制剂反应更差^[37]^。目前对该现象的原因还没有统一的说法，可能是因为在*EGFR*突变患者的肿瘤微环境中PD-L1表达过低和CD8阳性淋巴细胞较少，也可能是因为*EGFR*突变患者多表现非炎性表型和较低的免疫原性^[62, 63]^。此外，有研究^[64]^报道了肿瘤突变负荷（tumor mutational burden, TMB）与NSCLC中抗PD-1/PD-L1药物的ORR是呈线性正相关，另一项研究^[41]^则表示*EGFR*经典突变和20外显子插入突变的TMB是相似的，并且都低于EGFR野生型患者，这就说明*EGFR*罕见突变患者同经典突变患者相似，对免疫抑制剂治疗也不敏感。

另外，首次研究EGFR-TKIs联合抗血管生成抗体治疗*EGFR*突变患者的Ⅱ期临床试验^[65]^显示，与TKIs单药治疗相比，TKIs联合贝伐单抗治疗的中位PFS更长（中位PFS：16.9个月*vs* 9.7个月）。之后的Ⅲ期临床试验^[61, 66]^同样证实了该观点的正确性，结果显示，TKIs联合贝伐单抗组比TKIs单药组的中位PFS更长且ORR更高（中位PFS：16.9个月*vs* 13.3个月; ORR：72.3% *vs* 66.1%）。然而，上述试验中并不包括*EGFR*罕见突变患者，且暂时缺乏有关*EGFR*罕见突变与抗血管生成抗体的疗效关系的临床数据。想要进一步了解不同治疗方式对*EGFR*罕见突变患者的疗效，则需要更多的临床试验去探究*EGFR*罕见突变患者是否能从不同治疗方式中获益，以及如何用药才能获得最好的收益。由于罕见突变发生率低，选择EGFR-TKIs以外治疗方式的情况又少，所以这将是一项漫长的工作，我们要注意收集这部分患者的资料，并保证资料的客观性、真实性和完整性，相信未来*EGFR*罕见突变患者会获得更好的生存结果。

## 9 小结与展望

随着第三代TKIs的不断探索和发展，携带*EGFR*罕见突变的NSCLC患者的一线治疗方案可能会被改变，首选EGFR-TKIs治疗的患者会更多，甚至对前两代TKIs发生耐药的患者也可以继续应用第三代TKIs治疗。EGFR-TKIs治疗伴*EGFR*突变的NSCLC患者的前景非常可观，但对第三代TKIs发生耐药后的后续治疗仍是一个挑战，继续寻找新的治疗靶点，研制新一代靶向药物具有重要意义。由于*EGFR*罕见突变患者样本量少且具有高度异质性，EGFR-TKIs对*EGFR*罕见突变患者的疗效仍然不清楚。不过，多年来大量的临床研究表明，TKIs治疗携带*EGFR*罕见突变的NSCLC患者的疗效存在差异，且差异很大，提示我们在临床研究中应该对这部分患者进行单独分析，并为他们提供更有效的个体化治疗。虽然目前基因检测技术的发展日新月异，但临床上大部分的罕见突变仍不能被识别。当务之急就是尽快研发并使用能够更准确地识别不同*EGFR*罕见突变的基因检测方法，并以大型的前瞻性临床研究为依据，为*EGFR*罕见突变患者制定合理有效的治疗方案，取得更好的预后。
